# Radiomics-based analysis of CT imaging for the preoperative prediction of invasiveness in pure ground-glass nodule lung adenocarcinomas

**DOI:** 10.1186/s13244-022-01363-9

**Published:** 2023-02-03

**Authors:** Hui Feng, Gaofeng Shi, Qian Xu, Jialiang Ren, Lijia Wang, Xiaojia Cai

**Affiliations:** 1 Department of Radiology, The Fourth Hospital of Hebei Medical University, No. 12 of Health Road, Shijiazhuang, 050011 China; 2 GE Healthcare China, Beijing, 100176 China

**Keywords:** Radiomics, Lung adenocarcinoma, Ground glass opacity, Tomography (X-Ray Computed), Pulmonary nodules

## Abstract

**Objective:**

The purpose of the study is to investigate the performance of radiomics-based analysis in prediction of pure ground-glass nodule (pGGN) lung adenocarcinomas invasiveness using thin-section computed tomography images.

**Methods:**

A total of 382 patients surgically resected single pGGN and pathologically confirmed were enrolled in the retrospective study. The pGGN cases were divided into two groups: the noninvasive group and the invasive adenocarcinoma (IAC) group. 330 patients were randomly assigned to the training and testing cohorts with a ratio of 7:3 (245 noninvasive lesions, 85 IAC lesions), while 52 patients (30 noninvasive lesions, 22 IAC lesions) were assigned to the external validation cohort. A model, radiomics model, and combined clinical-radiographic-radiomic model were built using the LASSO and multivariate backward stepwise regression analysis on the basis of the selected and radiomics features. The area under the curve (AUC) and decision curve analysis (DCA) were used to evaluate and compare the model performance for invasiveness discrimination among the three cohorts.

**Results:**

Three clinical-radiographic features (including age, gender and the mean CT value) and three radiomics features were selected for model building. The combined model and radiomics model performed better than the clinical-radiographic model. The AUCs of the combined model in the training, testing, and validation cohorts were 0.856, 0.859, and 0.765, respectively. The DCA demonstrated the radiomics signatures incorporating clinical-radiographic feature was clinically useful in predicting pGGN invasiveness.

**Conclusions:**

The proposed radiomics-based analysis incorporating the clinical-radiographic feature could accurately predict pGGN invasiveness, providing a noninvasive biomarker for the individualized and precise medical treatment of patients.

## Key points

- Radiomics have potentials to differentiate the invasiveness of pGGNs lung adenocarcinoma.
- Clinical-radiographic feature adds discriminative value to radiomics in pGGNs pathological subtype.
- Combined clinical-radiographic-radiomic model can successfully stratify patients into noninvasive pGGNs and invasive pGGNs in patients with resectable lung adenocarcinoma.

## Introduction

Lung cancer is one of the most common and serious causes of cancer-related deaths worldwide [1]. With the development of high-resolution computed tomography (CT) and the popularization of low-dose CT screening, the incidence of pure ground-glass nodules (pGGNs) is rapidly increasing [2, 3]. A pGGN can be defined as a nodule with a hazy attenuation increase in the lung window, without a solid component when viewed with mediastinal window settings, and without vessel and bronchial structure obscuring [4, 5]. According to the new classification proposed by the International Association for the Study of Lung Cancer, the American Thoracic Society, and the European Respiratory Society, lung adenocarcinomas include atypical adenomatous hyperplasia (AAH), adenocarcinoma in situ (AIS), minimally invasive adenocarcinoma (MIA), and invasive adenocarcinoma (IAC) [6]. Persistent pGGNs lasting for more than three months have been proven to be associated with early stage lung adenocarcinoma including AAH, AIS, MIA and IAC [7–11]. Most pure GGNs are preinvasive lesions; however, recent histologic studies have shown that approximately 20% to 50% have invasive components [12–15]. In recent study, Ye et al. found that 10.8% of pure ground-glass lung adenocarcinoma nodules were of the IAC subtype among 988 pulmonary nodules [16].

Lobectomy is the standard surgical treatment for IACs; however, AIS and MIA may be candidates for sublobar resection [17]. Previous studies have demonstrated that the 5-year disease-free survival (DFS) of patients with AIS and MIA can reach 100% or almost 100%, while the DFS of patients with IAC is 40–85% [18]. Therefore, distinguishing IACs from preinvasive lesions and MIAs before surgery is crucial for clinical management and prognosis prediction in patients with pGGNs.

Clinical features including age, sex, and smoking history have been found as predictors of nodule growth and pathologic diagnosis [8, 19, 20]. Adenocarcinoma also accounts for a large percentage of lung tumors in female patients [21]. Huang et al. found that non-smoking female patients with lung cancer were more likely to have adenocarcinoma [22]. IAC occurred more often in older patients; Hu et al. found that aged ≥ 60 years was one of independent predictors of pGGNs histologic invasiveness [23].

CT is the most commonly used technique for the detection and differentiation of pGGN invasiveness [24]. Previous studies have shown that IAC differentiation is mainly based on radiographic features, including morphological features (margin, shape, vessel change, bubble sign, and pleural indentation) and quantitative features (lesion size, CT value, and volume) [14, 15, 19, 20, 25, 26]. While morphological features widely depend on the experience of observers and quantitative features are affected by scanning parameters (e.g., consistency of measurement), some features in pGGNs overlap; this is especially true in small nodules with a size of < 6 mm [27].

Radiomics that can extract high-throughput data from medical images and analyses with numerous quantitative descriptors in order to investigate the associations between imaging features and various endpoints have a promising potential for the evaluation of pGGN invasion [28–30]. Most previous studies have only extracted texture features from nonenhanced CT images and have included a small number of lesions. It would be very helpful in deciding on the optimal treatment plan if using clinical, radiographic and radiomics features for the differentiation of IAC from MIA and preinvasion (AAH and AIS) in evaluating pGGNs.

Therefore, the authors of the present study hypothesized that the combination of clinical, radiographic and radiomics features can improve the diagnostic ability to determine the histological invasiveness of adenocarcinomas appearing as pGGNs. The purpose of this study is to develop a combined prediction model in order to help guide an individualized preoperative design of surgical procedures.

## Materials and methods

### Study population

The present retrospective study was approved by the institutional research board, and the requirement for informed consent was waived.

**Fig. 1:**
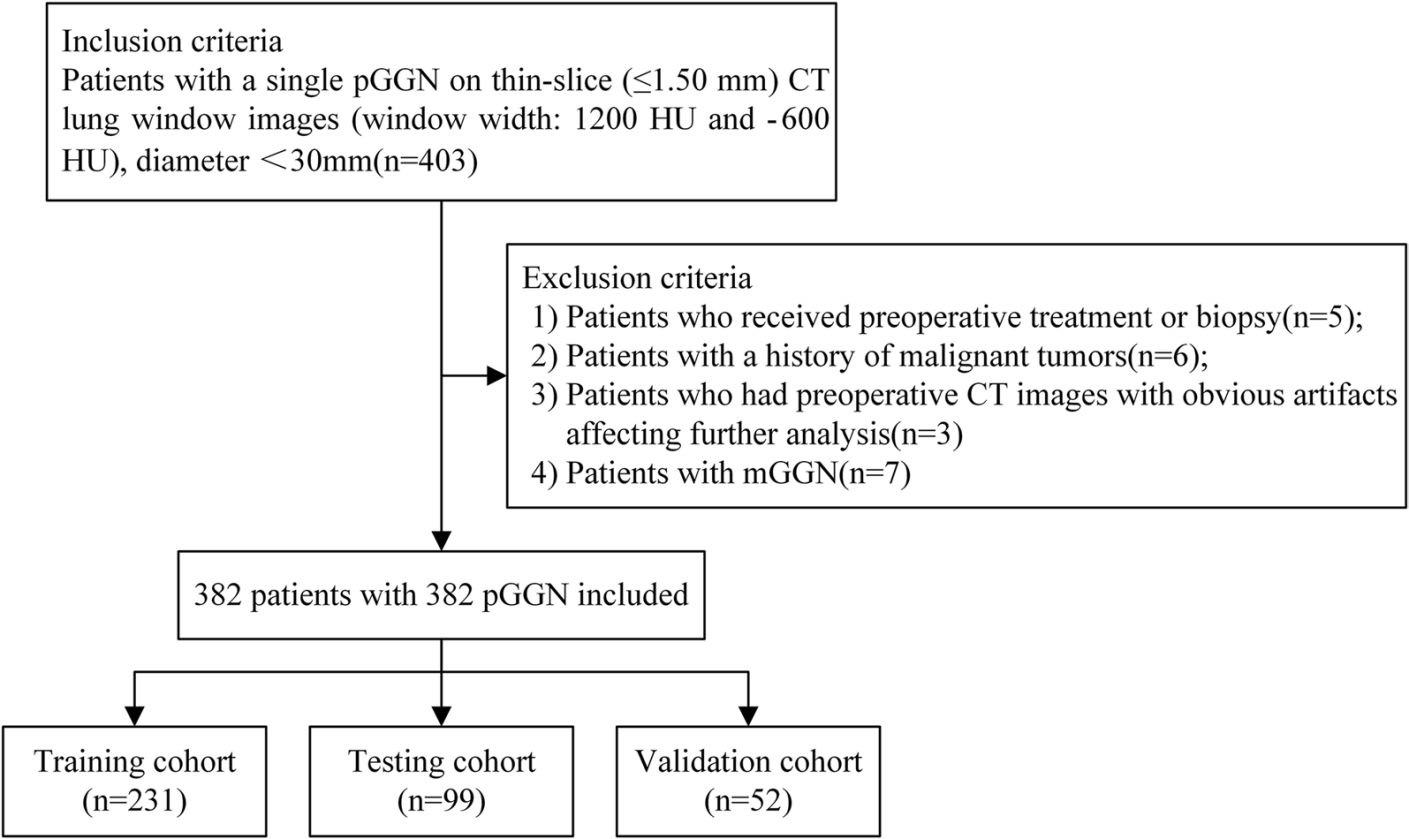
The flowchart of study population

The records and images of patients with pGGNs who underwent a preoperative chest CT were retrospectively reviewed. All nodules were pathologically confirmed as lung adenocarcinomas (AAH, AIS, MIA, and IAC) via surgical resection between January 2017 and December 2020 (Fig. 1 shows the flowchart of study population), lobectomy resection for patients with IAC, whereas limited resections for patients with AAH, AIS or MIA.

Inclusion criteria: (1) Patients with a single pGGN on thin-slice (≤ 1.50 mm) CT lung window images (window width: 1200 HU and −600 HU); (2) patients with a nodule diameter of < 30 mm; and (3) patients who underwent surgical resection (including lobectomy resection and limited resections) within one week of receiving a CT scan.

Exclusion criteria: (1) Patients who received preoperative treatment or biopsy which hemorrhage or exudates occurred around pGGNs; (2) patients with a history of malignant tumors; (3) patients who had preoperative CT images with obvious artifacts affecting further analysis, the decision was made by an experienced radiologist before grouping; and (4) patients with multiple GGN (mGGN).

Finally, 382 patients aged 35–73 years (mean age = 56 ± 10 years) with a total of 382 pGGNs were enrolled in the present study. The patients comprised 123 males aged 35–67 years (mean age = 54 ± 11 years) and 259 females aged 38–73 years (mean age = 58 ± 13 years). Of these patients, 231 were assigned to the training cohort and 99 were assigned to the test cohort randomly assigned to the training and testing cohorts with a ratio of 7:3; meanwhile, 52 patients scanned at the third CT were assigned to the external validation cohort. According to the pathological results (AAH, AIS, MIA, and IAC), preinvasive adenocarcinoma and MIA were considered noninvasive lesions. All included pGGNs were divided into two groups: the non-IAC group (*n* = 275; 72%) and the IAC group (*n* = 107; 28%).

### Acquisition of CT imaging

All patients received a contrast-enhanced chest CT using the Siemens SOMATOM Definition Flash, Siemens SOMATOM Sensation Open CT or GE Revolution. The detailed scanning parameters are listed in Table 1. The contrast-enhanced CT was performed after 25 s of intravenous administration of iodinated contrast material (2 mL/kg) at a rate of 3 mL/sec. The CT scans were acquired from all patients in the supine position at full inspiration. Scan coverage was from the lung base to the thoracic inlet.

**Table 1:** CT scanning parameters

Setting	Siemens SOMATOM definition flash	Siemens SOMATOM sensation open CT	GE revolution
Tube voltage (kV)	120	120	120
Tube current (mA)	35 mAs	35 mAs	Smart mAs
Pitch	1.2	1.2	1
Matrix	512 × 512	512 × 512	512 × 512
Slice thickness (mm)	1.0	1.0	1.25
Reconstruction algorithm	B60	B60	STND
Window width (HU)	1200	1200	1200
Window level (HU)	−600	−600	−600

### Image preprocessing

To minimize noise and processing artifacts, resampling of image data was avoided wherever possible, as previously described in similar investigations [31]. First, linear interpolate was applied to re-sampled the CT images and make voxel isotropic of 1 × 1 × 1 mm. Second, the image was discretized in gray scale and the binwidth was set to 25. Third, a Laplacian of Gaussian convolution kernel filter (*σ* = 3, 5, and 7) and wavelet transform was employed to decrease noise and enhance features at different spatial scales.

### Clinical characteristics and CT image evaluation

According to the research literature related to lung cancer [32], the clinical characteristics, including age, gender, smoking status, and clinical symptoms, were derived from medical records. Two thoracic radiologists with 5 and 10 years of experience, respectively, reviewed the CT images of each patient and identified the radiographic features including morphological and quantitative features without knowledge of the patient’s pathological results. Decisions regarding the CT features were reached by consensus. Morphological features included lobulation, spiculation, pleural indentation, and the vacuole sign. Quantitative features included the lesion maximum diameter and mean CT value. The CT images were read with a lung window setting (width: 1200 HU; level: −600 HU).

### Reproducibility analysis

To ensure reader reproducibility, 50 patients were randomly selected for a reproducibility analysis. For the evaluation of the interobserver agreement of the radiomics features, two radiologists with 8 years (Reader 1) and 10 years (Reader 2) of experience in chest CT interpretation, respectively, completed the region of interest (ROI) delineation without any information regarding the patients. Next, Reader 1 repeated the ROI delineation with an interval of one week for the assessment of the interobserver agreement. The interobserver and interobserver agreement of the radiomics feature extraction were evaluated using interclass and intraclass correlation coefficients (ICC). Features with an ICC of > 0.8 were regarded as an acceptable agreement and were included in the subsequent analyses.

### Segmentation of pulmonary nodules

Segmentation of pulmonary nodules was performed on the thin-slice enhanced images with a lung window setting (width, 1200 HU; level, −600 HU) using the ITK-SNAP software (version 3.8.0, https://www.itksnap.org). The radiologist delineated the nodule length, and the software automatically drew regions of interest covering the entire range of the tumor on the axial CT images. The radiologist manually adjusted and identified the boundary regions on each section. The involved pleura, blood vessels, and bronchi at the edge of the nodules were excluded from nodule segmentation.

### Radiomics feature extraction

Radiomics feature extraction was performed using the PyRadiomics. A total of 1130 radiomics features were extracted from the contrast-enhanced CT image for each patient [33]. The extracted features comprised tumor shape features, first-order statistic features, and texture features, such as the gray-level co-occurrence matrix (GLCM), gray-level run length matrix, gray-level dependence matrix, gray-level zone matrix (GLSZM), and neighborhood gray tone difference matrix, to reflect internal heterogeneity as previously described [34].

### clinical-radiographic-feature-based model

Relevant clinical features, including sex, age, smoking history, and clinical symptoms, and radiographic features including morphological and quantitative features were analyzed to build clinical-radiographic-based model.

The clinical feature selection was performed in three steps: (1) The Shapiro–Wilk test was used to test the normality of the data sets; (2) the Student t test or Wilcoxon rank sum test was used for the continuous variables (mean CT value and maximum diameter), and the ${\chi }^{2}$ or Fisher exact test was applied for the categorical variables (sex, smoking history, and clinical symptoms); and (3) the stepwise multivariable logistic regression analysis was applied to obtain the independent clinical risk factors and build a clinical-feature-based model.

### Radiomics-feature-based model

The radiomics-feature-based model was built in four steps: (1) The correlation analysis was performed to identify the redundant features (features with a correlation coefficient of > 0.8 were eliminated) [28]; (2) the Mann–Whitney U-test was performed to compare the differences between each radiomics feature in the two groups (radiomics features with a *p* value of < 0.05 were kept); (3) the least absolute shrinkage and selection operator (LASSO) was performed for feature selection [35–38]; and (4) the stepwise multivariate logistic regression analysis based on the Akaike information criteria was performed to identify the optimal radiomics features for the differentiation of noninvasive lesions and IACs. Then, a combined predictive model was built with the use of selected conventional and radiomics features.

The performance of the three models in the training, testing, and validation cohorts was evaluated by the receiver operating characteristics (ROC) curves; the area under the curve (AUC), accuracy, sensitivity, and specificity were calculated, respectively. A radiomics signature-based score (Rad-score) for each outcome was then obtained from the final model. The Rad-score cut-off values for differentiation of the non-IAC group and the IAC group were chosen according to the Youden index criteria. To estimate model goodness-of-fit, calibration curves were performed, and the Hosmer–Lemeshow test was used to assess model consistency [39, 40]. Decision curve analyses (DCAs) were used to evaluate the potential net benefit based on the clinical diagnosis, radiomics, and the combined model in the different cohorts.

## Results

### Patient demographic characteristics

A total of 382 patients with pGGNs were enrolled in the present retrospective study according to the inclusion and exclusion criteria. Among them, 275 were diagnosed with noninvasive lesions (AAH, AIS, and MIA) and 107 were diagnosed with IAC. The training cohort comprised 330 patients (330 pGGNs); these patients were divided into the training cohort and the testing cohort according to the ratio of 7:3 with stratified sampling. A total of 52 patients (52 pGGNs) were assigned to the validation cohort. The demographic characteristics of three cohorts of the 382 patients are listed in Table 2.

**Table 2:** Demographic characteristics

	Training cohort			Testing cohort			Validation cohort		
	NonInvasion	Invasion	*p* value*	NonInvasion	Invasion	*p* value	NonInvasion	Invasion	*p* value*
	*N* = *172*	*N* = *60*		*N* = *73*	*N* = *25*		*N* = *30*	*N* = *22*	
Gender			0.041			0.032			0.107
Female	124 (72.1%)	34 (56.7%)		54 (74.0%)	12 (48.0%)		17 (56.7%)	18 (81.8%)	
Male	48 (27.9%)	26 (43.3%)		19 (26.0%)	13 (52.0%)		13 (43.3%)	4 (18.2%)	
Age	56.0 [47.0; 62.0]	59.5 [53.8; 64.0]	0.016	55.0 [48.0; 61.0]	59.0 [54.0; 64.0]	0.110	57.0 [52.0; 64.0]	60.5 [53.5; 65.0]	0.295
Center			0.302			1.000			0.317
Absent	84 (48.8%)	24 (40.0%)		32 (43.8%)	11 (44.0%)		19 (63.3%)	10 (45.5%)	
Present	88 (51.2%)	36 (60.0%)		41 (56.2%)	14 (56.0%)		11 (36.7%)	12 (54.5%)	
AxisMaxLength	1.02 [0.80; 1.24]	1.53 [1.19; 1.95]	< 0.001	1.01 [0.84; 1.30]	1.56 [1.17; 1.97]	< 0.001	1.29 [0.95; 1.67]	1.28 [1.18; 1.63]	0.453
Distance	0.99 [0.29; 1.82]	0.74 [0.00; 1.56]	0.193	0.68 [0.29; 1.49]	0.66 [0.00; 1.20]	0.427	0.50 [0.00; 1.71]	1.27 [0.59; 1.86]	0.187
Burr			0.001			0.001			1.000
Absent	170 (98.8%)	53 (88.3%)		71 (97.3%)	18 (72.0%)		27 (90.0%)	20 (90.9%)	
Present	2 (1.16%)	7 (11.7%)		2 (2.74%)	7 (28.0%)		3 (10.0%)	2 (9.09%)	
Lobe			0.001			0.014			0.299
Absent	170 (98.8%)	53 (88.3%)		72 (98.6%)	21 (84.0%)		29 (96.7%)	19 (86.4%)	
Present	2 (1.16%)	7 (11.7%)		1 (1.37%)	4 (16.0%)		1 (3.33%)	3 (13.6%)	
Vacuole			0.224			0.050			1.000
Absent	163 (94.8%)	54 (90.0%)		72 (98.6%)	22 (88.0%)		29 (96.7%)	21 (95.5%)	
Present	9 (5.23%)	6 (10.0%)		1 (1.37%)	3 (12.0%)		1 (3.33%)	1 (4.55%)	
Pleura Involve			0.017			0.255			1.000
Absent	172 (100%)	57 (95.0%)		73 (100%)	24 (96.0%)		29 (96.7%)	22 (100%)	
Present	0 (0.00%)	3 (5.00%)		0 (0.00%)	1 (4.00%)		1 (3.33%)	0 (0.00%)	
MeanCT	−562.50 [−634.72; −480.93]	−501.25 [−545.38; −413.53]	< 0.001	−508.80 [−588.00; −407.60]	−471.90 [−567.20; −408.10]	0.544	−597.55 [−638.65; −551.20]	−493.10 [−566.88; −391.80]	< 0.001
Clinical			0.497			0.855			0.475
Absent	130 (75.6%)	42 (70.0%)		53 (72.6%)	17 (68.0%)		23 (76.7%)	14 (63.6%)	
Present	42 (24.4%)	18 (30.0%)		20 (27.4%)	8 (32.0%)		7 (23.3%)	8 (36.4%)	
Smoke			0.071			0.004			0.161
No	146 (84.9%)	44 (73.3%)		66 (90.4%)	16 (64.0%)		22 (73.3%)	20 (90.9%)	
Yes	26 (15.1%)	16 (26.7%)		7 (9.59%)	9 (36.0%)		8 (26.7%)	2 (9.09%)	

*p value is derived from statistical analyses between each of variables and groups. *p* value < 0.05 indicated statistical significance. Values are presented as no. (%) or mean (95%CI). Chi-square test or Fisher’s exact test was used for the categorical variable. A Student’s *t* test, Mann–Whitney *U*-test or Kruskal–Wallis *H*-test were used for the continuous variable

### Feature selection

According to the univariate analysis results, three clinical features (gender, age, and smoking history), six radiographic features including four morphological features (burr, lobe, vacuole sign, and pleural involvement), and two quantitative features (mean CT value and axis max length diameter) were found to be significant in the differentiation between noninvasive lesions and IPAs in the training cohort; only one quantitative feature (mean CT value) was found to be significant between noninvasive lesion and IPAs in the external validation cohort.

According to multivariable logistic regression, age, gender, and the mean CT value were selected to build clinical-radiographic-based model. After LASSO and the stepwise logistic regression analysis were conducted, three radiomics features were ultimately selected to build a radiomics-feature-based model. These features included the Log.5.0_glszm_SmallAreaHighGrayLevelEmphasis, wavelet.LHL_glcm_MCC, and wavelet.LLL_glcm_SumAverage.

### Performance of clinical-radiographic, radiomics, and combined models

**Fig. 2:**
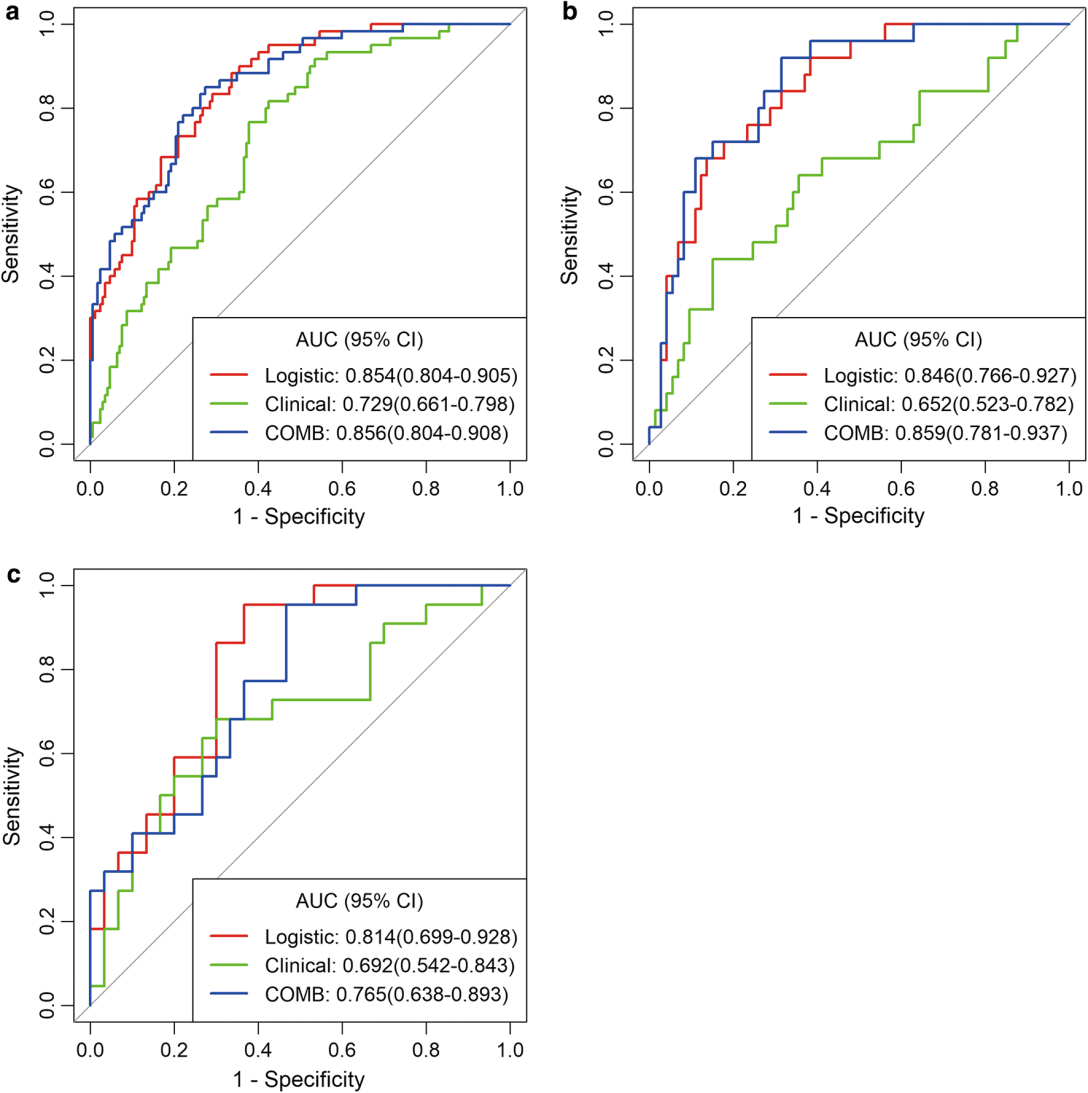
The ROCs of the three models in the training cohort (**a**), testing cohort (**b**), and validation cohort (**c**). The predictive performance for an invasive pGGN lesion was better in the combined model than in the clinical and radiomics models in the training and testing cohorts. In the validation cohort, the radiomics model and combined model performed better than clinical model

The ROCs and AUCs of the three cohorts are shown in Fig. 2A–C. The best model was the combined model in the training and testing cohorts, with an AUC of 0.856 and 0.859, in the validation cohort, the radiomics model and the combined model performed better than clinical model, with an AUC of 0.814, 0.765 and 0.692, respectively. The radiomics models showed an excellent predictive performance in the discrimination of IACs from noninvasive lesions in the three cohorts. The radiomics signature yielded an AUC of 0.854, 0.846, and 0.814 in the training, testing, and validation cohorts, respectively (Table 3), and the sensitivity was 0.883, 0.840 and 0.955, specificity was 0.663, 0.644 and 0.633 in three cohorts.

**Table 3:** Diagnostic performance of three models for the prediction of pGGN invasiveness

Cohort	Model	AUC	95% CI	Sensitivity	Specificity	Accuracy
Training cohort	Clinical-radiographic model	0.729	0.661–0.798	0.817	0.576	0.638
	Radiomics model	0.854	0.804–0.905	0.883	0.663	0.720
	Combined model	0.856	0.804–0.908	0.850	0.727	0.759
Testing cohort	Clinical-radiographic model	0.652	0.523–0.782	0.680	0.452	0.510
	Radiomics model	0.846	0.766–0.927	0.840	0.678	0.729
	Combined model	0.859	0.781–0.937	0.880	0.685	0.735
Validation cohort	Clinical-radiographic model	0.692	0.542–0.843	0.682	0.600	0.635
	Radiomics model	0.814	0.699–0.928	0.955	0.668	0.769
	Combined model	0.765	0.638–0.893	0.773	0.656	0.762

95% CI means 95% confidence interval

**Fig. 3:**
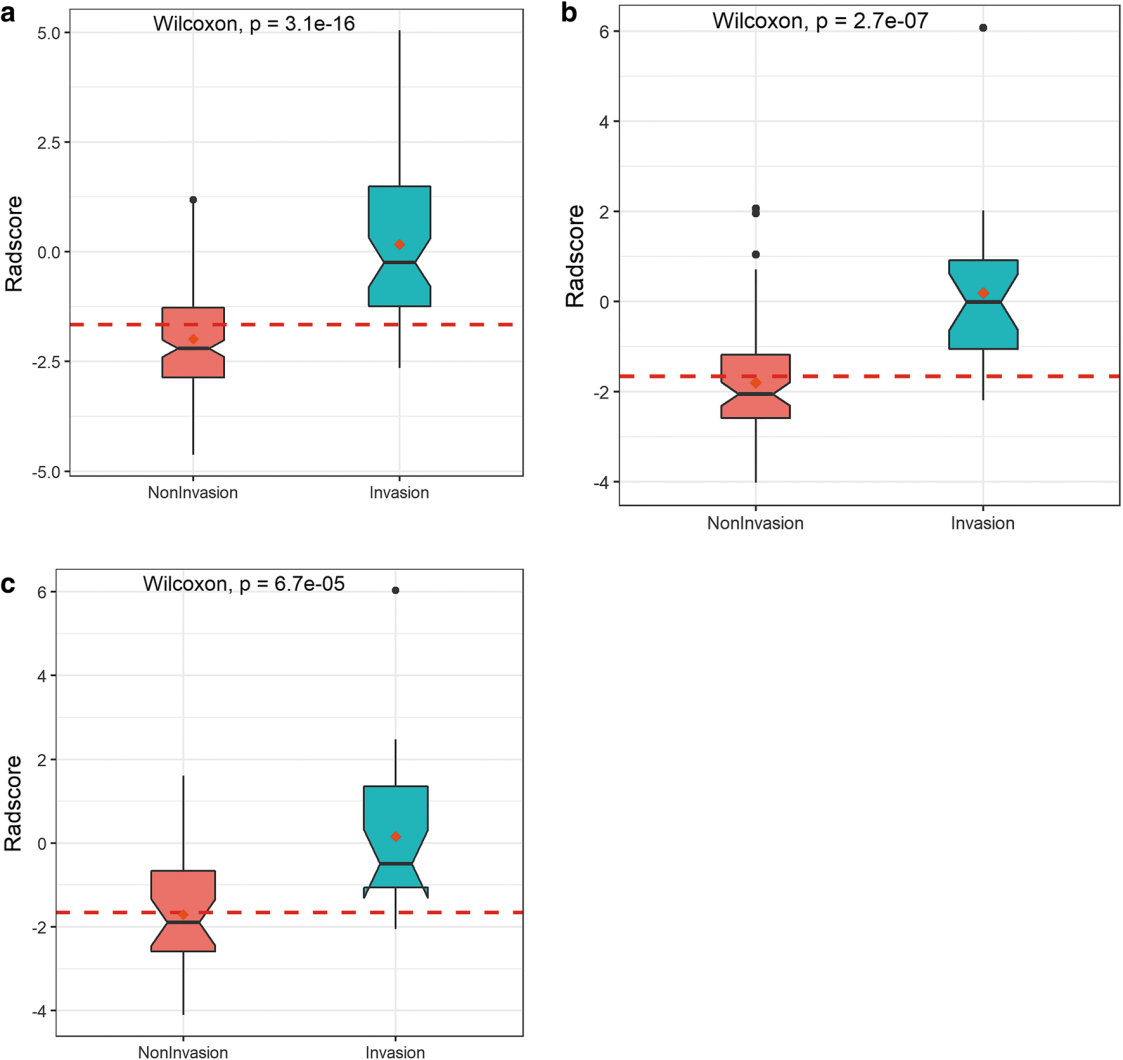
The box plots show the distribution between noninvasive and invasive lesions for GGNs in the training cohort (**a**), testing cohort (**b**), and validation cohort (**c**). The Wilcoxon test obtained *p* values. The Rad-score was higher in the invasive group than in the noninvasive group

The Rad-score was calculated from the selected radiomics features. The corresponding regression coefficients and distribution in the training, testing, and validation cohorts are presented in Fig. 3. The Rad-score cut-off value was −1.66.

**Fig. 4:**
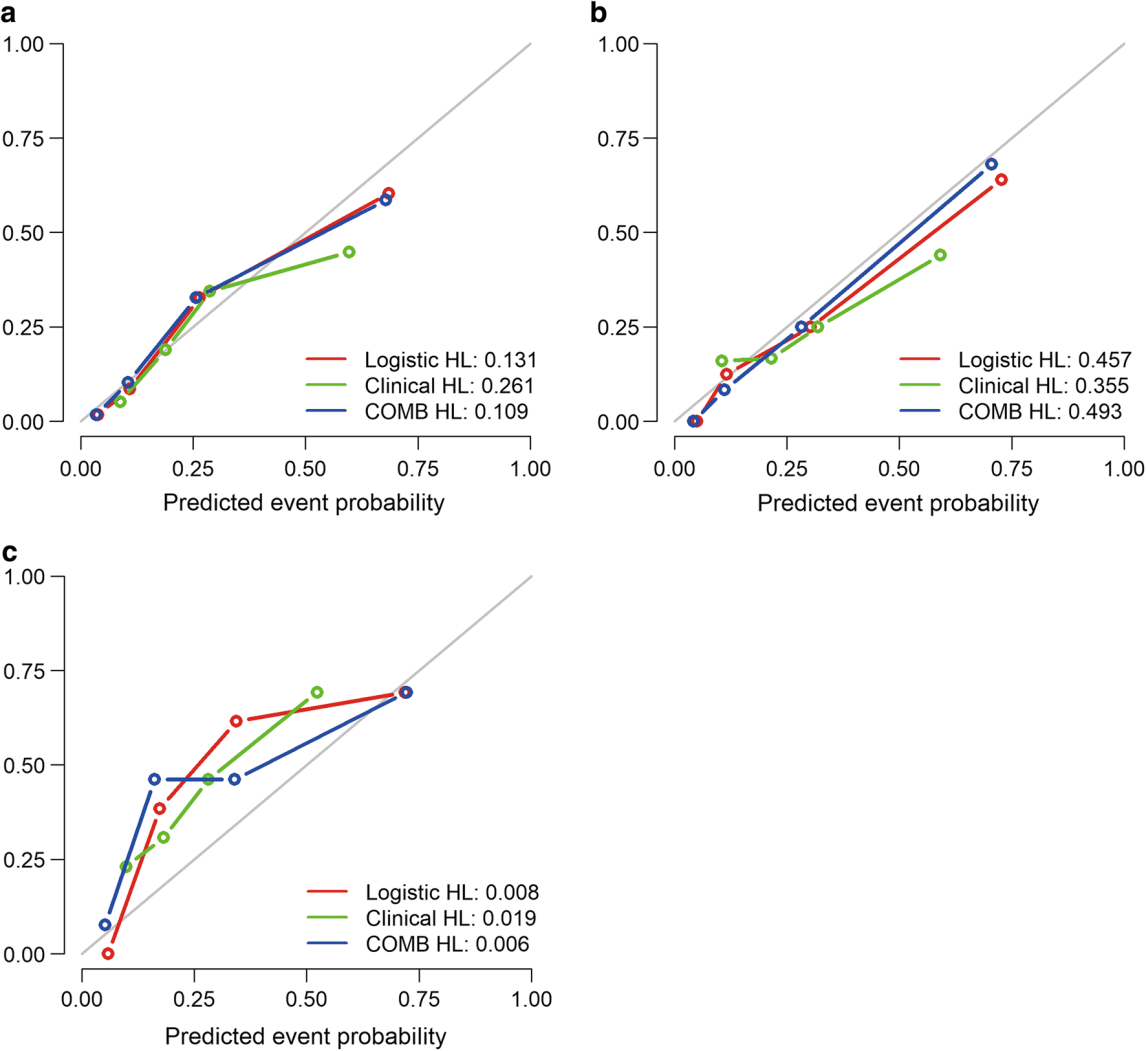
Calibration curves for the prediction of pGGN invasiveness based on the three models in the training cohort (**a**), testing cohort (**b**), and validation cohort (**c**). The x-axis represents the predicted probability of IACs based on the clinical, radiomics, and combined models, and the *y*-axis represents the actual probability of pGGN invasiveness. The 45° diagonal line represents ideal prediction, and the red, green, and blue lines represent the predictive performance of the nomogram. The closer the line was to the ideal line, the better the predictive nomogram performance

The calibration curves showed good predictability between prediction and observation in the three cohorts (Fig. 4). Compared with the results of the validation cohort, the correspondence between actual and ideal predictions suggested good calibration of the clinical, radiomics, and combined models in the training and testing cohorts.

**Fig. 5:**
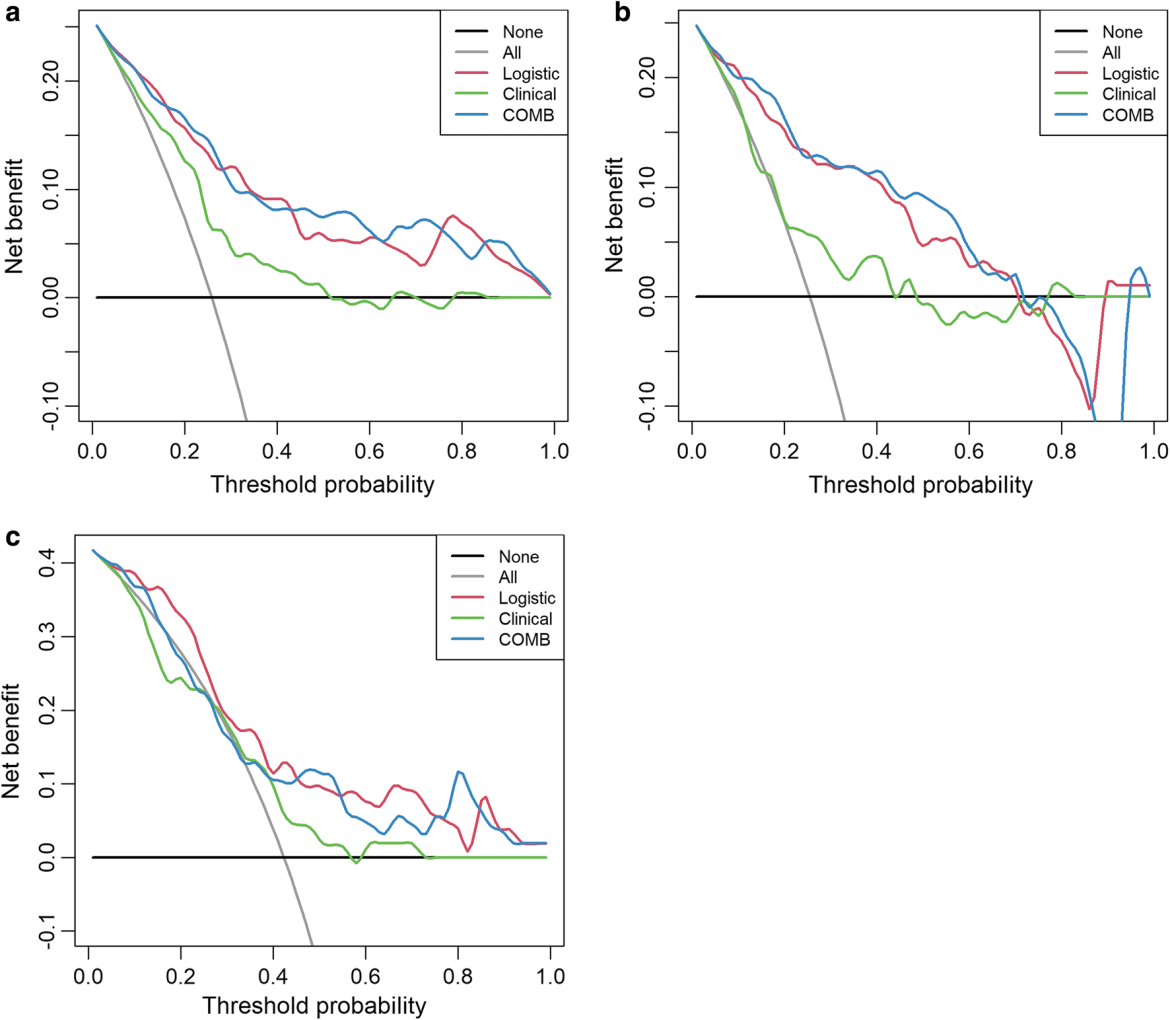
Decision curve analysis for the combined model (**c**) compared with the clinical model (**a**) and radiomics models (**b**) alone. The *x*-axis shows the threshold probability, and the y-axis measures the net benefit. The black line represents the hypothesis that all patients with pGGNs had noninvasive lesions, and the gray line represents the hypothesis that all patients with pGGNs had invasive lesions

The decision curve analysis for the three models is presented in Fig. 5. The decision curve indicated that the use of a combined model for the prediction of invasive lesions added more net benefit than the use of clinical features or radiomics features alone in the differentiation of IACs from noninvasive lesions; this was especially true in the training and testing cohorts. The DCA in the external validation cohort showed that the combined and radiomics models achieved higher average precision scores than the clinical-radiographic model.

Clinical-radiographic features, including gender, age, mean CT value, and radiomics features (Log.5.0_glszm_SmallAreaHighGrayLevelEmphasis, wavelet.LHL_glcm_MCC, and wavelet.LLL_glcm_SumAverage), were significantly associated with an increased risk of IACs. The highest odds ratio (OR) was detected for gender in the clinical model (OR = 2.466), for wavelet.LLL_glcm_SumAverage in the radiomics model (OR = 2.287), and for wavelet.LLL_glcm_SumAverage in the combined model (OR = 2.471) (Table 4).

**Table 4:** Associations between features and invasiveness of pGGNs

	OR	95% CI	*p* value
Clinical model			
Gender	2.466	1.266–4.860	0.0083
Age	1.037	1.033–1.074	0.0356
Mean CT value	1.006	1.003–1.009	0.0000
Radiomics moel			
Log.5.0_glszm_SmallAreaHighGrayLevelEmphasis	2.232	1.481–3.441	0.0001
wavelet.LHL_glcm_MCC	0.579	0.370–0.884	0.0132
wavelet.LLL_glcm_SumAverage	2.287	1.528–3.549	0.0001
Combined model			
Log.5.0_glszm_SmallAreaHighGrayLevelEmphasis	2.201	1.446–3.419	0.0002
wavelet.LHL_glcm_MCC	0.603	0.382–0.929	0.0247
wavelet.LLL_glcm_SumAverage	2.471	1.623–3.928	0.0000
Gender	2.190	1.004–4.832	0.0491

## Discussion

Radiomics are a rising field of quantitative imaging that can extract high-throughput data features and describe tumor phenotype characteristics. Radiomics analysis provides a noninvasive and powerful alternative for disease diagnosis, differentiation, clinical treatment, and assessment [34]. Recent studies have succeeded in radiomics analyses in the field of oncology, showing the diagnostic and prognostic values [34, 41, 42].

Therefore, in the present study, the radiomics features and evaluated clinical features were extracted from CT images for the prediction of pGGN invasiveness; they were then investigated, and the performance of the clinical, radiomics, and combined models for classifying noninvasive lesions and IACs was compared.

The results of the present study showed that the combined model performed better than the radiomics and clinical models, with a higher AUC in the training and testing cohorts (0.856, 0.859, respectively), suggesting that it is a noninvasive tool for differentiating IACs from noninvasive lesions.

Some studies have suggested that radiomics features can be used to differentiate pGGN invasiveness. Previous studies reported that in clinical practice, lesion size, CT value, and morphological characteristics were associated with pGGN invasiveness [43–45]. Recent studies have highlighted the combined model, which incorporated clinical features and radiomics features in the diagnosis of lung cancer.

Sun et al. and Liu et al. [46, 47] showed that the predictive model for IAC constructed by integrating the clinical and radiomics features based on the radiomics nomogram exhibited excellent accuracy in the differentiation of noninvasive lesions from IACs (AUC 0.831; 95% CI: 0.765–0.897). Although morphological characteristics, such as lobulation, burr, vacuole sign, and pleural involvement sign, are helpful in the identification of nodule invasiveness, they were not included in the final clinical model construction of this study; this is mainly because the morphological characteristics of early stage lung cancer are usually atypical, especially in IACs with a smaller diameter than 1 cm. Furthermore, identification of the morphological features depended on the radiologist’s diagnostic experience.

Former studies have found that the mean nodule CT value is a significant predictor in the differentiation of pGGN invasiveness. Previous study has shown that pGGNs with a mean CT value higher than −600 HU indicated invasive adenocarcinoma [23].

Wu et al. [48] evaluated CT and histopathologic features of lung adenocarcinoma with pGGNs that were ≤ 10 mm in diameter, and the results showed no statistically significant difference in the CT value between noninvasive lesions and IACs.

In the present study, the multivariate logistic regression analysis revealed that the mean CT value was useful in histopathologic subtype differentiation; IACs reflected a higher mean CT value than noninvasive lesions. The study also showed that the AUC for the clinical model established by using gender, age, and the mean CT value was lower than the AUC for the radiomics model in the three cohorts (AUC = 0.729, 0.652, and 0.692, respectively).

Radiomics show the ability to serve as a bridge between medical imaging and precise medicine [49–51]. Yang et al. [52] utilized 14 radiomics features of lung adenocarcinoma to distinguish IACs and noninvasive lesions; an AUC of 0.77 was achieved. The radiomics signatures performed better than the most commonly used clinical features, such as the mean CT value. Weng et al. [53] identified that the nomogram, which integrated morphology characteristics and radiomics features, showed a high performance in the classification of IACs and MIAs (AUC = 0.888).

In the present study, the radiomics features performed a good differentiation ability in three cohorts (AUC = 0.854, 0,846, and 0.814, respectively); this ability was significantly better at predicting IACs than in the clinical model. The findings of the present study are consistent with the findings of previous studies, which have found various texture and shape features to be significant predictors of IACs.

Furthermore, radiomics features comprising tumor shape features, first-order statistic features, and texture features in a noninvasive, three-dimensional manner may allow for more precise and personalized treatment than traditional modality of these patients with pGGN detected by CT.

The clinical and radiomics models were then combined to improve the diagnosis accuracy; the combined model had a better performance than clinical and radiomics model alone and achieved a satisfactory result in the external validation cohort. The ROC and DCA also indicated that using the combined model to predict invasive lesions added more net benefit than using clinical features or radiomics features alone in differentiating IACs from noninvasive lesions, especially in the training and testing cohorts.

The present study has several limitations. First, it is a single-institutional retrospective study, and the sample size is small; large and multi-institutional cohorts will be recruited in future research. Second, the CT images were acquired from different scanners, and standardization of scanning and reconstruction parameters is required for further study. Third, although an external validation cohort was constructed, the number of patients assigned to it was relatively small; thus, it is necessary to increase the number of cases in this group to identify the performance of the model and usefulness of radiomics signatures.

## Conclusion

In conclusion, the radiomics signature from the CT images provided a noninvasive modality for IAC prediction. Radiomics signatures combined with clinical features yielded a better performance than using alone in differentiating IACs from noninvasive lesions appearing as pGGNs on thin-slice CT; this may facilitate clinical diagnosis and treatment in further work.

## Abbreviations

pGGNs: Pure ground glass nodules
IAC: Invasive adenocarcinoma
LASSO: Least absolute shrinkage and selection operator
AUC: Area under the curve
DCA: Decision curve analysis
HRCT: High-resolution CT
AAH: Atypical adenomatous hyperplasia
AIS: Adenocarcinoma in situ
MIA: Minimally invasive adenocarcinoma
DFS: Disease-free survival
CT: Computed tomography
ICC: Intra-class correlation coefficients
GLCM: Gray-level co-occurrence matrix
GLRLM: Gray-level run length matrix
GLDM: Gray-level dependence matrix
GLSZM: Gray-level zone matrix
NGTDM: Neighborhood grey tone difference matrix
AIC: Akaike information criterion
